# Acceptability of a Mobile-Health Living Kidney Donor Advocacy Program for Black Wait-Listed Patients

**DOI:** 10.3390/ijerph18168239

**Published:** 2021-08-04

**Authors:** John C. Sieverdes, Lynne S. Nemeth, Martina Mueller, Vivik Rohan, Prabhakar K. Baliga, Frank Treiber

**Affiliations:** 1 Department of Health and Human Performance, College of Charleston, Charleston, SC 29424, USA; 2 College of Nursing, Medical University of South Carolina, Charleston, SC 29424, USA; nemethl@musc.edu (L.S.N.); muellerm@musc.edu (M.M.); treiberf@musc.edu (F.T.); 3 College of Medicine, Medical University of South Carolina, Charleston, SC 29424, USA; rohanv@musc.edu (V.R.); baligap@musc.edu (P.K.B.)

**Keywords:** kidney, transplantation, disparities, mHealth

## Abstract

Marked racial disparities exist in rates of living donor kidney transplantation (LDKT). The Living Organ Video Educated Donors (LOVED) program is a distance-based, mobile health program designed to help Black kidney transplant wait-list patients advocate for a living donor. This study reported on the acceptability outcomes to aid in future refinements. Participants were randomized to LOVED (*n* = 24, mean age = 50.9 SD (9.2) years), male = 50%) and usual care groups (*n* = 24 (mean age 47.9 SD (10.0), male 50%). Four LOVED groups completed an eight-week intervention that consisted of six online video education modules and eight group video chat sessions led by a Black navigator. Qualitative analysis from post-study focus groups resulted in six themes: (1) video chat sessions provided essential support and encouragement, (2) videos motivated and made participants more knowledgeable, (3) connectivity with tablets was acceptable in most areas, (4) material was culturally sensitive, (5) participation was overall a positive experience and (6) participants were more willing to ask for a kidney now. The video chat sessions were pertinent in participant satisfaction, though technology concerns limited program implementation. Results showed that the LOVED program was acceptable to engage minorities in health behavior changes for living donor advocacy but barriers exist that require future refinement.

## 1. Introduction

The burden of end-stage renal disease (ESRD) in the United States reached an all-time high in 2018 with an unadjusted incidence of 390.2 cases per million people [1]. Out of the nearly 660,000 people with ESRD, approximately 90,000 patients were on the UNOS kidney transplant wait-list. Even though transplantation is acknowledged as the best treatment for ESRD patients, total transplants have stabilized over the last decade, with 22,816 kidney transplants performed in 2020. When stratified by deceased and living donor kidney transplants (LDKTs), there are notable trends. In the last five years, proportions of LDKTs have decreased from 31.5% in 2015 to 22.9% in 2020 [2]. A more concerning fact is the marked disparity between Black and White LDKT recipients during the same time frame. The prevalence of LDKTs in White patients was 44.9% (3696/8226) in 2015 and decreased to 33.0% (*n* = 3367/10,217) in 2020, whereas the prevalence for Black patients was 13.2% (*n* = 662/5015) in 2015 and reduced to 9.5% (*n* = 595/6233) in 2020 [2]. Proportions worsen when accounting for geographical differences, especially in the southeastern United States (i.e., Region 3 Southeastern US data for Black LDKT prevalence = 5.5% is half the national rate; Region 1 (New England area): 2020 data prevalence = 20.3% is double the national rate.) [2,3]. Therefore, increased attention is warranted to intervene with minority populations to increase LDKTs, especially in the southeastern US.

LDKT promotion is a focus for several US governmental agencies including the National Institutes of Health and other nonprofit organizations such as the National Kidney Foundation’s “The Big Ask: The Big Give”. Recent efforts to reorganize organ procurement policies have improved the utilization of available kidneys since December 2014 [4], but additional interventional programs are needed to enhance LDKT, especially in various ethnicities and races [5]. Behavioral interventions intended to enhance LDKTs need cultural tailoring and to be widely disseminated to overcome individual barriers to enhance patients’ support systems, knowledge and skills [6,7,8]. Prior evidence has identified many barriers in Black ESRD patients to identify potential donors (PDs). These include a lack of knowledge to answer questions (e.g., financial misconceptions of the donor having to pay for surgery, confusion on who qualifies to be a donor, risks of surgery, etc.) and myths about kidney transplantation (e.g., religious misconceptions, more likely to become an ESRD patient if one donates a kidney, health consequences of the donor, etc.) [5,9,10,11,12,13]. Of particular importance, the barriers to overcoming feelings to ask others and lacking relevant skills to develop personal strategies are critical to find a PD.

Addressing the barriers of Black kidney wait-list candidates raises concerns about being able to provide programs to those with few resources. There have been several key LDKT programs that support patients, often using a partner or group sessions to enhance behaviors to advocate for an LDKT, specifically the Donor Champions and Kidney Coach programs [14,15]. Although effective in increasing donor inquiries, these programs relied on in-person interactions for training which may not reach patients in the most need. Barriers such as finding times to meet, location requirements, parking and travel time result in additional burdens on patients who already suffer from debilitating fatigue and dialysis schedules. Utilizing mobile health (mHealth) technology is a potential method to resolve this issue and provide education and behavioral change opportunities for patients while limiting logistical concerns. The use of mobile devices has increased dramatically, where 81% of the US population and 80% of the Black population had a smartphone in 2019 [16].

To address the LDKT disparity, the Living Organ Video Educated Donors program (LOVED) was developed to overcome barriers and provide an easily accessible LDKT promotion program using mHealth technology to deliver education videos and group video chat sessions with a Black navigator who underwent an LDKT [17]. The purpose of the program was to improve the knowledge and skills of Black kidney wait-listed patients to approach PDs about their need. This report discusses the acceptability of the LOVED program through focus group evaluations and the assessment of cultural competency using qualitative and quantitative measures on trust, discrimination, decision making and communication. Results provide insight on the benefits and shortcomings of the intervention, which could aid in developing future transplant center programs using distance-based approaches to address LDKT disparities.

## 2. Methods

### 2.1. Study Design

The LOVED program was developed using a three-phase, iterative-design strategy [17]. As a disparities-focused intervention, the target population was Black kidney transplant wait-listed patients. Phase one used a qualitative approach using focus groups comprised of medical providers, Black living donors, Black kidney recipients and their family members to develop the initial program concept and create the LOVED application [6,9]. Phase two used a 1-arm, proof-of-concept trial [18] with successive iterative refinement from focus group feedback. The third phase used a 2-arm randomized feasibility design with survey measures and post-study focus groups [19]. The primary outcomes for the third phase and expanded description of the protocol are published elsewhere [19]. In brief, the primary outcomes focused on (1) program tolerability measured using attrition rates, (2) program fidelity measured by completion rates of video education modules, attendance to the online video chat sessions and (3) change in LDKT attitudinal and knowledge questionnaires. Secondary outcomes reported transplant center data including the number of screening calls, LDKT evaluations, and completed LDKTs between study arms [19]. This article reports the tertiary acceptability outcomes from the feasibility trial including participant perspectives pertaining to the LOVED app’s usability, program topics, group and navigator interactions, positive and negative aspects of the trial and insight into any experiential behavior changes. Although phase three consisted of participants randomized to LOVED and usual care arms, the qualitative results were limited to the LOVED arm. Complete details of the LOVED phases, theoretical development, measures and qualitative methodology are explained in detail elsewhere [17]. The Consolidated Criteria for Reporting Qualitative Research (COREQ) 32-item checklist was used in the development of this report [20]. All study procedures were approved by the institution’s internal review board and included written informed consent prior to participants’ involvement in research activities (IRB#: Pro00031456). The trial was registered with clinicaltrials.org (NCT03599102) and conducted between August 2016 and September 2017.

### 2.2. LOVED Intervention

LOVED is a distance-based behavioral change education program designed to aid Black kidney wait-list candidates advocate for an LDKT. Using theoretical constructs from the Self-Determination Theory [21] and Social Cognitive Theory [22], the program was developed with a user-centered approach. The intervention’s aim was to progressively build knowledge, skills and enhance self-efficacy in participants to approach others about becoming a PD. The 8-week program consisted of baseline and post-study survey measurements with follow-up surveys performed at six months. Focus groups were conducted post-intervention at week 8. The entirety of the LOVED program, including informed consent, was performed through email, phone contact, and video chat conferencing. Three primary elements integral to the LOVED intervention included: (1) weekly computer tablet-delivered, web-based educational video materials, (2) weekly peer-navigator-led video chat discussions, and (3) optional discussion and communications between group members and peer-navigators to provide social support and additional strategy sessions. The LOVED app was developed at the Medical University of South Carolina and used a HTLM-5 based web-app program formatted to be viewed through a tablet computer interface. The app’s interface consisted of an introductory and six weekly education sessions. Each session consisted of three short videos (e.g., 2–4 min in length) with 1–3 quiz questions presented to the participant after each video to assess understanding. Additional resources specific for each session’s topic leveraged existing transplant center information, additional testimonials, news clips and kidney organization websites. A backend server captured all app usage data including logins, quiz attempts and each video participants completed. The research staff maintained access to these data through a web-based portal to communicate group completion rates and quiz scores results to the navigators on a weekly basis.

The education content was structured using weekly topics to enhance knowledge, dispel myths and build rapport with other group members during the first four weeks (i.e., 3 video education modules (12 total videos) and 4 video chat sessions). The final four weeks’ content focused on skill development, practice asking others during video chats sessions and brainstorming individual strategies to initiate discussions with PDs (i.e., 3 video education modules (11 total videos) and 4 video chat sessions) Participants were also encouraged to self-initiate identifying and speaking to PDs [19]. Strategies included direct methods such as asking PDs individually, in groups and indirect methods such as email, social media (e.g., Facebook, Twitter, etc.) and other forms of media (e.g., newspaper, television, car wraps, etc.).

The LOVED and usual care groups both received the standard transplant center education program prior to being wait-listed. This included an in-person 3 h classroom-based seminar on topics ranging from deceased and LDKT options, post-transplant costs such as medications, fundraising and required medical testing for ESRD patients and PDs. Patients’ physicians also advocated for living donation during regular transplant center appointments. Lastly, a living donor coordinator was assigned to each participant to answer questions about LDKT as part of standard care.

An important component of this culturally tailored intervention was the use of Black LDKT recipients as the program’s navigators to increase the relatedness between the educator and the participant. This decision was reaffirmed by the focus groups’ results from phase one and two. The navigators participated in a 7 h training session with research staff on the program content, weekly discussion questions, program delivery, participant contact and reporting outcomes. Two college-educated Black men in their 40s were identified and chosen to be navigators through prior involvement in advocacy efforts with the transplant center. A modest stipend was paid for their contribution.

### 2.3. Participants

Potential participants were identified from the state’s single transplant center’s active wait-list database. Inclusion criteria consisted of identifying as Black race, being wait-listed for five years or less, able to speak and read English, 18 to 65 years of age and having 10 or less years of dialysis treatment including pre-emptive patient status. This combination of inclusion criteria was chosen by the research team through expert opinion for high medical viability in patients to have a successful LDKT. Exclusion criteria consisted of acute or chronic mental health conditions identified by each patient’s transplant physician that would limit their ability to complete the program, or already having a PD in the living donation process. A list of potential participants was cleared by their transplant physicians and contacted by a transplant center coordinator to discuss the LOVED trial. Research staff followed up with those who showed interest and were mailed informed consent documentation. After consent documents were delivered, research staff discussed the study details by phone. Participants who agreed signed and returned the informed consent by mail.

### 2.4. Sample Size

The sample sizes used in this study were chosen for pragmatic reasons since the purpose of phase three was to conduct a feasibility trial. A modest sample goal of 30 persons per arm was used due to the limited number of tablet computers and resources available.

### 2.5. Protocol

Randomization was conducted using a random number generator. After allocation, tablet computers (i.e., iPad Air 2 (Apple Inc., Cupertino, CA, USA)) were mailed to each LOVED arm participant followed by phone contact to complete tablet setup and individualized training for 30 min. This included information about the study, surveys and explanation of introductory tutorial videos that included instructions on how to navigate the apps, Wi-Fi connections (i.e., preferred method of connection) and charging. Baseline surveys were administered to each participant using electronic surveys (i.e., REDCap, (Vanderbilt University, Nashville, TN, USA)) or mailed hard copies approximately one week prior to the orientation video chat session. The purpose of the orientation video chat session (i.e., using VidyoMobile App (Enghouse Vidyo Inc., Hackensack, NJ, USA)), between the navigator and group members was to perform introductions and set regularly scheduled meetings. During the subsequent eight weeks, group members were instructed to watch the weekly assigned video education modules (e.g., 10–15 min per week) prior to the weekly video chat sessions (e.g., 30 min to 1 h). A variety of discussion topics about the education videos were planned for each video chat session to reinforce knowledge on LDKT. In addition, strategies, stories and skills would be discussed so the navigator and group members could share their perspectives, barriers and successes. Participants were sent reminders to complete the video education sessions each week using email and text messaging depending on their preference by research staff. Reminders also included a list of videos that had not been completed. Reminders to attend the video chat sessions were sent one day prior as well as 10 min prior to the beginning of the meeting. Those missing video chat sessions were contacted for an explanation why they were absent.

Prior to the last session, participants were asked if they would volunteer to attend a post-study focus group immediately after the last scheduled video chat session. The navigator left prior to the focus group due to questions concerning feedback on their role in the study. The focus groups were scheduled for 30–45 min, led by a Ph.D. level researcher trained in qualitative research and designed to elicit feedback on the LOVED program components (i.e., the behavioral changes participants experienced, perceptions on their attitudes, and the role of the navigators). Each participant attending the focus groups was compensated USD 25 for their time. All participants received USD 50 after each completed survey for baseline, 8-week and 6-month measures.

### 2.6. Measures

Post-study acceptability measures were used to assess the cultural competency of the program using both quantitative and qualitative methods and included questionnaires at 8 weeks and 6 months. The “Commonwealth Fund’s Culturally Competency and Quality of Care: Using the Patient’s Perspective” was used as a framework to identify and assess the intervention’s components [23]. These included the following four valid instruments.

Adapted from AHRQ’s Medical Expenditure Panel Survey [24], the Communication Effectiveness Questionnaire measured communication perspectives (e.g., Do your health providers, LOVED navigator and research staff listen to you? Do they explain things in a way that is easy to understand? Do they show respect for what you have to say?) between the participant and the navigator, research staff and renal transplant team. Communication was scored using the average of a 9-item Likert-type scale (range 1–4), with 4 representing stronger communication.

The Abbreviated Trust Questionnaire measured how much trust the participants had toward research staff during the conduct of the study (e.g., I trusted the information in the program, the advantages and disadvantages of having a transplant were explained to me, etc.) [25]. Trust was scored using the average of a 3-item Likert-type scale questionnaire (range 1–4), with 4 representing more trust.

The Experiences of Discrimination Questionnaire is a valid and reliable self-report measure to identify psychological distress from racism and inequity due to exposure to an experience, with our example being the LOVED program. Scores used the average of a 5-item Likert-type scale (range 1–4), with 4 representing less perception of discrimination (e.g., As a participant in the LOVED program, did you feel: discriminated against because of your race, income, where you live, etc.) [26]. A low score indicates perceptions or actual negative interactions that are based on gender, race, ethnicity or other demographic factors by the study staff.

The Shared Decision Making Questionnaire (SDM-Q-9) measured perspectives of decision making between the participant and LOVED Navigator (e.g., I felt like I was involved in making a decision about transplantation. I was able to ask about different treatment options while in the program, etc.) [27]. It was scored using the average from a 6-item Likert-type scale (range 1 to 4), with 1 representing stronger empowerment on making their own decision.

### 2.7. Data Analyses

#### 2.7.1. Quantitative Analyses

Feasibility outcomes were compared between usual care and LOVED study arms. Baseline characteristics were analyzed using means, standard deviation (SD), frequencies with group comparisons using ANOVA and Chi-Square tests reporting means with 95% confidence limits. Post-study questionnaires were evaluated with ANOVA if statistical assumptions for equal variance were met or Welch–Satterthwaite tests if assumptions were not met. Data were analyzed using SAS Statistical Software Version 9.4 (Copyright© 2016 by SAS Institute Inc., Cary, NC, USA) and SPSS Statistics, Version 25.0 (IBM Corp, Armonk, NY, USA).

#### 2.7.2. Qualitative Analyses

A qualitative expert developed the interview content using results from the LOVED proof-of-concept study and suggestions from two navigators to gain feedback on education content, skill-based elements and program delivery perceptions. The initial development of focus group scripts followed the guidelines from The Survey Kit [28]. Content areas included app usability, video chat sessions, video education modules, length and time commitment and interactions with the navigators and study staff. Table 1 reports the focus group script and prompts. Research staff conducted the four LOVED groups directly after each of the final scheduled video chat session. Focus groups were not performed for the usual care group. Recordings from the post-study focus groups were then transcribed and evaluated using NVIVO 10 (QSR International Pty, Doncaster, Victoria, Australia) by one qualitative expert. A process of inductive analysis, based on grounded theory, was used to categorize and structure each group’s comments into similar meanings [29,30]. Using open coding structure, each transcript was analyzed line-by-line to identify segments of key text without preconceived thematic categories [30]. In combination with the focus group questions, this allowed the generation and refinement of themes regarding being a LOVED participant. Although the total number of focus groups was limited to four, saturation was determined as met by the qualitative analyst. The themes and coding structure were then reviewed by two authors, where non-verbal cues in transcript data were integrated using a process of immersion. The method of crystallization was used for confirmation of the resultant themes, where all sources of data were systematically reviewed by the two authors until agreement for the final themes [31].

## 3. Results

### 3.1. Sociodemographic Variables

**Figure 1:**
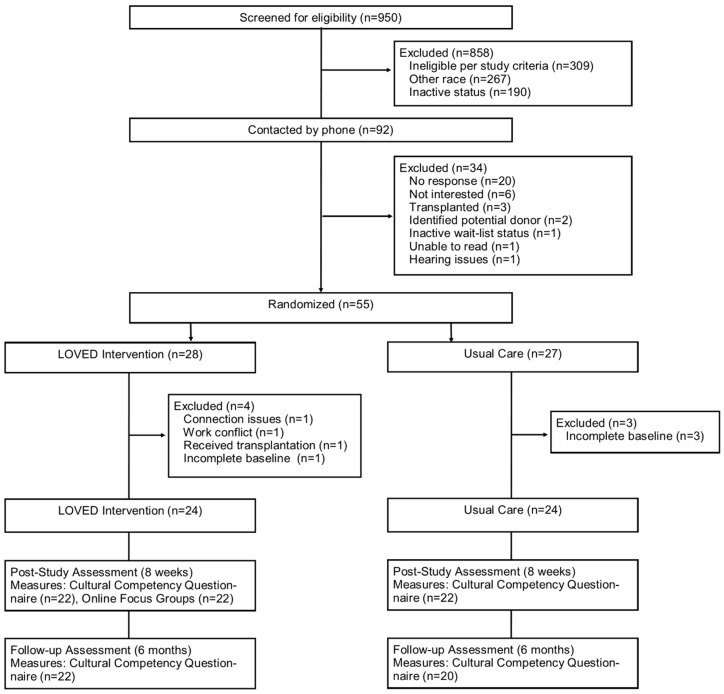
Participant flow chart.

Illustration of the flow of participants, including measures and sample sizes, is found in Figure 1. Of the 92 eligible patients, 55 completed the informed consent and were randomized to LOVED (*n* = 28) and usual care (*n* = 27) arms. Post-randomization, four LOVED arm participants did not start the study and were excluded due to persistent tablet connection issues, work conflicts, received a transplant and failed to complete the baseline questionnaire. Three usual care participants did not complete the baseline questionnaire and were also excluded. Two participants left the study in the LOVED arm, leaving a sample of 22, while the usual care group had 2 nonresponders at eight weeks and 4 nonresponders at six months. No statistically significant differences were observed in sociodemographic variables (all *p*’s ≥ 0.29) between study arms (see Table 2). The LOVED group was predominately middle aged (mean age = 50.9 years SD (9.2), with half being females. A majority of the participants held a technical school or college degree (62.5%) and were not employed (66.7%). The usual care group averaged 47.9 SD (10.0) years old and half were females, with the same number of participants having a technical school or college degree (62.5%). Four LOVED groups were created that consisted of equal numbers of men and women, with six to eight participants each.

### 3.2. Summary of Primary and Secondary Findings

Although the trial’s primary and secondary findings have been published elsewhere, a brief summary is included to support our findings [19]. Program tolerability was found to be high, with 95.8% retention, though fidelity was found to need improvements, with only 78.9% of the videos being completed and with 72.1% video chat adherence. Compared to usual care, the LOVED arm had statistically significant improvements in willingness to approach strangers (*p* = 0.009) about living donation, positive changes in self-efficacy (*p* < 0.001) with other attitudinal scores favoring the LOVED arm. Secondary outcomes showed improvements in transplant center results including higher PD inquiries (*p* = 0.008) compared to the usual care arm, though no differences were found for transplant center evaluations. No LDKTs were completed during the trial period, but one LOVED participant did enter the paired donor exchange program during the study by six months [19].

### 3.3. Thematic Findings

The primary results from this report involve the results from the qualitative analysis. Six themes were identified and included: (1) video chat sessions provided essential support and encouragement, (2) videos motivated and made participants more knowledgeable, (3) connectivity with tablets was acceptable in most areas, (4) material was culturally sensitive, (5) participation was overall a positive experience and (6) participants were more willing to ask for a kidney now. Table 3 depicts these sentiments through exemplar quotes.

#### 3.3.1. Theme: Video Chat Sessions Provided Essential Support and Encouragement

A strong aspect of this program involved the ability to speak with others about their kidney disease beyond gaining knowledge through video education modules. Participants shared their successes, frustrations, and feelings throughout the program during the video chat sessions. This created a safe space to vet new ideas, form relationships, and provided a group that related to each other’s struggles in finding a living donor.

#### 3.3.2. Theme: Videos Motivated and Made Participants More Knowledgeable

Video education modules were well received as a component in the program. Participants found value in the videos to gain knowledge about the transplant process and prepare them for the discussion topics during the video chat sessions. Participants noted overcoming several myths about PD concerns such as risk to the donor’s remaining kidney, donor perspectives, the donation surgical process and introducing several strategies to aid them in their search. Participants had an overall positive perception on the order and timing of the material, including the weekly assignments and limited burden watching the material.

#### 3.3.3. Theme: Connectivity with Tablets Was Acceptable in Most Areas

A strong broadband or cellular signal was suggested as necessary when watching the video education modules and when participating in the video chat sessions. Participants said connectivity improved when tablets were connected to their home Wi-Fi network. Cellular connectivity was available for every tablet through a 3G LTE connection, though there were instances of dropped video chat calls, video module buffering, and inability to connect to the video chat session in a few homes. Several participants were able to establish a better connection by relocating to another location during the video chat sessions. Two participants opted to use their own tablet computer. There was agreement that the LOVED program was best watched on a screen larger than a smartphone so individual group members could be seen during the video chat sessions. Research staff held troubleshooting sessions with participants if issues persisted or participants were unable to join or stay connected to the video chat sessions.

#### 3.3.4. Theme: Material Was Culturally Sensitive

Participants unanimously felt that the LOVED content was culturally sensitive and included elements that focused on Black patients. Participants mentioned that they were aware LOVED was for Black patients though they raised the thought that other racial groups should be welcome to participate. Participants noted that since kidney disparities are strong in their area of the country, groups would predominantly be made up of Black patients. On the topic of if navigators should be Black, participants responded that the gender or race of the navigators did not matter as much, but it was very helpful to connect with someone who had lived with kidney disease and successfully identified a living kidney donor.

#### 3.3.5. Theme: Participation Was Overall a Positive Experience

All participants reported having an overall positive experience in the LOVED trial. Several group members were planning joint activities to advocate for a PD by the end of the trial. Focus groups participants mentioned being encouraged and motivated by their navigator. There were many mentions about the benefit of increasing knowledge and reducing their personal barriers when asking others for a kidney. Overall, participants did not feel the program was too short or too long. Responders elaborated that the format and time commitment of the video education app were appropriate and the video chat sessions were very important to enhance their skills and provide motivation. Even though the value of the video education modules was evident, there was a general consensus that participants looked forward to the video education sessions more to interact with the navigators and other group members.

#### 3.3.6. Theme: More Willing to Ask for a Kidney Now

The participants noted speaking to others about their need for a kidney. Most of the one-on-one interactions were between the patient and extended family members rather than reaching out to co-workers, those involved in organizations (i.e., religious organizations) or strangers. The comfort level with family was evident across all focus groups irrespective of gender. Reponses also indicated that by the end of the LOVED intervention, most had already asked all of their family and were strategizing how to approach them for follow-up.

### 3.4. Participant Perspectives on the LOVED Navigators

The participants had many positive comments about having a navigator lead the video chat sessions. The navigators were trained to interact with each of the participants and gave weekly assignments in alignment with the weekly video module such as creating an elevator speech, asking others to be an advocate for them, bringing up living donation to a stranger or identifying their support network. Focus group responses supported this notion.

“He kept it interesting. He always um bring some good to the table or give us like homework that we can have something to discuss when we come back together.”

“He kept us engaged, and he didn’t just allow you to sit back and not participate. Um he drew you in even when you didn’t want to answer, you had to say something.”

“The format was good because he uh, he been through the process I’m going through so any advice that he gave helped me cross that bridge in my process so…the way it was laid out, the way he gave it was good.”

### 3.5. Quantitative Findings with Qualitative Integration

Questionnaire findings (see Table 4) were used to assess the trial’s cultural competency. The LOVED group reported high communication scores (mean = 3.93 out of 4 (95% CI: 3.86, 3.99)) at 8 weeks compared to usual care (mean = 3.78 (95% CI: 3.62, 3.93)), with both groups exhibiting very high scores. This trend continued at 6 months. Many participants mentioned communication was good, giving examples of text message reminders before video chat sessions. Several quotes from the focus groups related to communication exemplify other points.

“The staff have always been nice and helpful to me when I ask questions.”

“Everyone does a great job; everybody is patient and understanding with any question you have. Thank you for the program and [I] learned a lot.”

“My experience with [the facility’s] Transplant Team has been wonderful. However, there was a few times where there was miscommunication.”

The LOVED group specific questionnaire on trust demonstrated high mean scores for both 8-weeks (mean = 3.95 out of 4 [95% CI: 3.85, 4.05]) and at 6 months. Focus group comments also showed high levels of trust when speaking about their navigators, research staff, and medical staff.

“I have had two prior kidney transplants within a twelve-year period. The doctors and surgeons at [the facility] are very knowledgeable in their field. They perform hundreds of transplants per year. I trust them completely with my life.”

“He’s [speaking about their navigator] had that experience and he told us about stuff that he experienced. Which was great, because to hear somebody else talk about it, without them turning up their nose at you and saying, “oh, you have this”, and they don’t want to hear what you have or whatever, he wasn’t even like that. Not at all. He was great.”

The LOVED arm’s results on discrimination were very positive at 8 weeks (mean 3.96 out of 4 (95% CI: 3.89, 4.02)) and 6 months for the study staff during the trial. Scores did not differ compared to usual care for either follow-up time (8 weeks mean = 3.86 (95% CI: 3.71, 4.02)) or at 6 months). Many comments reinforced this idea. When asked if they thought whether the program was culturally sensitive, there was agreement that there was “no problem”.

“Me personally? I don’t have a problem with it [meaning discrimination]. But by the way things are looking, this is a disease that affects African Americans a lot, I mean because that basically who’s in this group.”

“For me it was helpful because I was blind to the fact. I thought because I’m a black man, that only a black person could donate a kidney to me, and I found out that’s not true.”

The LOVED-specific questionnaire on shared decision making was strongly skewed toward high levels of empowerment (mean = 1.33 out of 4 (95% CI: 1.01, 1.64) at 8 weeks, with lower scores representing a stronger sense of empowerment). These results corresponded to various comments from the focus groups, especially when participants spoke about their navigator.

“The format was good because he been through the process I’m going through so any advice that he gave helped me cross that bridge in my process so…the way it was laid out, the way he gave it was good.”

“And I really learned that from him. Because I’m just like, “I’m tired, I’m ready to move forward in my life, I’m just tired of waiting.” So, you know, I learned that from him. That is the most valuable thing that I can get from the program.”

## 4. Discussion

This study investigated the feasibility and acceptability of a mobile health interventional program for Black kidney wait-list patients to approach and ask others about being a PD. Results will inform refinement of LOVED and provide value to other transplant center programs in promoting culturally tailored strategies to increase living donations for Black ESRD patients. From the previous findings, the LOVED program was found to be well tolerated and improved quantitative measures in approaching others for a transplant. This was evident in the attitudinal measures and in donor inquiries when compared to usual care. The primary results of this report found six themes expanding upon the previous findings that explains how participants were either supported or hindered during the intervention. Overall, participants reported being satisfied with the program, being more confident about their knowledge on LDKTs and were more comfortable to ask others about being a PD. A key tactic suggested by the thematic results emphasized the importance of video chat sessions due to the mentorship role of the navigators and the encouragement from the group members. Comments were overwhelmingly positive when referring to content and discussions during the chat sessions, skill building, and comradery that ensued during the program. We found the design and elements of LOVED was successful to address the concerns of our sample. Participants also commented that the program could be inclusive of other races both in membership and navigator choice due to the strong bond of being a kidney disease patient overcoming the issue of race. In this sample, there were no issues of distrust or discrimination involving the navigators, research staff or their transplant team.

### 4.1. Discussion of Themes

The themes highlight several tactics that may enhance living donation programs. The theme, (1) Video chat sessions provided essential support and encouragement, was a crucial element in the delivery of the LOVED program. It is possible that LOVED could be performed solely using the video chat sessions if more time was allotted to go over the knowledge content. The emphasis on the video chat sessions from focus group feedback shows future iterations of the LOVED program should involve this intervention component. The theme, (2) videos motivated and made participants more knowledgeable, provided basic knowledge to be used in their discussions during the video chat sessions. The videos allowed the same knowledge to be communicated regardless of the navigator, standardizing much of the content across all groups. The reinforcement of video content by the navigator-led discussions allowed reiteration of the material and strengthened knowledge. Although the videos included motivational and skill building messages, the focus groups did allude to the video chat sessions being the primary factor to become more comfortable approaching others. Overall, the education videos were perceived as relevant and important to the intervention.

The theme related to technology, (3) connectivity with tablets was acceptable in most areas, was the primary area where improvements could be made. The technology aspects of this study should underscore the pace of technology changes in cellular network connectivity, as well as video chat applications used during the study timeframe. During the trial, we experienced many technical issues where participants could not connect to chat rooms, buffering and frozen video connections and participants not completing the questionnaires due to connectivity issues. Even though we provided 3G LTE connected tablets with unlimited data, throughput was not adequate to enable a seamless experience. As improvements in high-speed cellular services evolve (i.e., 5G and beyond, improved coverage, etc.) and next-generational video data compression algorithms become available, not only will connectivity improve, but participants may be able to utilize their own smartphone or tablet devices easier.

Pertaining to the theme, (4) the material was culturally sensitive, LOVED was designed utilizing focus groups of Black living donor kidney recipients, Black ESRD patients, and Black family members and caregivers. The culturally tailored aspect of this study was foundational in the LOVED videos and interventional content. This was evident in the race of the navigators, the education videos using predominantly non-White speakers, and using focus group feedback from prior studies. Our positive findings from the focus groups, trust and discrimination measures show that participants were receptive to the program content and staff.

The emphasis of the last two themes, (5) participation was overall a positive experience and (6) participants were more willing to ask for a kidney now, implied the LOVED program was successful in enacting the behavior change and attitudes toward finding a PD. Each of the four LOVED groups’ responses showed that overall, they felt less alone in their situation and started getting the word out. Focus group members reported having new hope in eventually identifying a PD. When prompted for what to change, several participants expressed desire to remain connected and have follow-up sessions. Most participants were happy with the content, the length of the program and the opportunity to connect with others who share their circumstances.

Although intentions to find a PD were voiced, no living donations were performed in the post-study, 6-month timeframe, though several participants had received deceased donations. Several barriers to finding a PD included running out of immediate or extended family members to ask and not being as confident speaking to friends or strangers. Comments also included that many family members lived unhealthy lives, had diabetes or were already sick with multiple chronic diseases. Lastly, many stated that obesity-related barriers were present in PDs that were interested in donating. It is likely that additional content or other programs based on common barriers may need to be incorporated in programs to recruit living donors. Overall, the participants showed motivation to ask others, but they were having issues identifying suitable donors.

### 4.2. Comparisons to Other Studies

This study shares several aspects with other living donor programs seeking to enhance LDKTs. We were able to replicate the findings from the LOVED proof of concept study, where patients reflected positively to develop social support, LDKT knowledge, and increased behavioral skills to seek a PD [18]. As with the prior study, the current study also found the chat and video education content helpful in learning how to approach PDs. A similar challenge was the connectivity of the device and connection issues of the video chat call. Other studies have used similar online education support such as the Explore Transplant at Home program using video education which saw increased intention of dialysis patients to engage in transplant programs [32]. The Talk About Live Kidney Donation (TALK) was also aimed at the ESRD community and used video education in addition to a visit by a social worker to increase LDKT behaviors [33]. These programs, aimed at Black clinical populations, all saw increased engagement in their respective behavioral outcomes similar to the LOVED program. Our study also agreed with the Donor Champions program, which was 5.8 times more likely to have a PD referral and increased their comfort in approaching others [14]. Another trial was the Kidney Coach program that used similar education tactics, which also increased donor inquiries compared to control (*p* = 0.001) [15]. Both programs were performed in-person, used skill building to improve communication between the patient’s team and PDs but relied on different types of educators. Overall, our findings were similar to other programs and support the notion that multiple methods can affect behaviors in Black ESRD patients regarding LDKT promotion.

### 4.3. Limitations

There are several limitations that should be discussed when interpreting the results. This trial was rigorously conducted at an academic medical center using Ph.D. level study staff. The navigators were college educated and have had experience promoting donation in their communities. Therefore, their level of enthusiasm, education, and medical center oversight may not be generalizable to community-based programs. It is also unknown whether the LOVED elements can be independently used due to the concurrent use of all study elements in this trial. Future studies are warranted using different combinations of the LOVED elements. The formative nature of these results limits the implications of the LOVED program on clinical outcomes for transplant centers. Primarily, changes in attitudes and behaviors may not directly result in successfully identifying a PD and completion of an LDKT. There are many complex factors related to the donor and the patient and that may limit the chance of a successful LDKT. Identifying these barriers will provide important information to include in a comprehensive program aimed at improving living donation rates in the southeastern United States and in the nation.

### 4.4. Future Considerations

Several themes suggest future improvements for the LOVED program. Primarily, the connectivity of the tablet computers was found in both the proof of concept and the current trial. The sample included patients from across the state, representing both rural and urban areas. Efforts to increase connectivity and problem-solving weak signals need to be emphasized in future studies or any video-chat-directed studies. Solutions could range from cellular extenders, hot spot devices and other packaged content for the education videos such as being stored locally rather than using streamed video content from a server. It is also reasonable to reformat the program to be utilized on participants’ smartphones instead of supplying tablets, which lowers the cost and administration burden of the program. Even though the length of the program was judged acceptable by the participants, there are data to support modification of program content to increase education video completion rates and video chat adherence. This may include shortening the program. Longer term contact with participants such as inclusion of booster sessions may also be considered to sustain the behavior changes elicited by the LOVED program.

### 4.5. Conclusions

The results of this study are timely considering the current pandemic and risk of holding in-person meetings at transplant centers or other community venues. The push to conduct education online has been an important consideration prior to this restriction due to ESRD patient barriers including transportation needs, burden of dialysis and conflicting schedules. This study found the LOVED program to be a feasible and acceptable method for Black kidney wait-list patients to get the word out about their need for an LDKT.

## Figures and Tables

**Table 1:** Focus group script.

**Overall program administration:** 1. How did you feel interfacing with the tablet computer that we provided and the programs we used for the LOVED program? 2. Did you use the tablet for anything else besides the program? 3. How reliable was the connectivity of the tablet for you? 4. What do you think about the overall program length and the time you had to commit? Prompt: Does anybody think it should be shorter or even longer? Prompt: How about the amount of time needed to complete the video education modules? 5. Would you want access to all the education videos at once at the beginning of the program, or do you like how we had it structured where the next set of videos opened after the current week’s video chat session? 6. What do you think about the time you were given to practice the skills taught in the last half of the program? 7. I wanted to ask your opinion about the number of video chat sessions we had. Did you meet enough times; too many? Do you need more flexibility on the meeting times? Tell me in general how you felt about this. **Video chat session element:** 8. Tell me your impressions about being a part of the video chat sessions. 9. Tell me what you thought about our choice of navigators. Prompt: What do you think their strengths are and what they could have done better? 10. Think back to the video chat sessions that you participated in. Which ones stood out as most useful? Prompt: Why is this so? Prompt: How about the sessions that may be the least useful? **Education module element:** 11. Now we are going to discuss the LOVED education modules and the video content. So overall, tell me what you felt about interacting with the tablet and watching the video education sessions. 12. From the quiz questions at the end of each video; what did you think of them? 13. Were there any set of videos that seemed to be the most useful for you? 14. Do you think the information in the videos were covered thoroughly enough? Prompt: Did topics need to be covered more in depth? 15. Do you think the information from the videos were sensitive to you as a Black man or woman? Prompt: How do you think the videos cater to you? 16. Now remember the extra resource links at the bottom of each weekly session? How helpful were they? **Impact of the Program** 17. Describe your overall impression about being in the LOVED program. Include how it changed you. 18. How did the LOVED program actually change your willingness to ask others for a kidney? 19. Was there anything else you were expecting in the LOVED program that was not covered? 20. Were there any resources you felt you still needed after the program had ended? Prompt: Resources? Additional support?

**Table 2:** Demographic characteristics.

Characteristics	LOVED (*n* = 24)	Usual Care (*n* = 24)	*p*-Value
Age, y [mean (SD)]	50.9 (9.2)	47.9 (10.0)	0.29
Sex (Male), [*n*, %]	12, 50.0%	12, 50.0%	>0.99
Marital status, [*n*, %] Lives alone Married or living with significant other	9, 37.5% 15, 62.5%	13, 54.2% 11, 45.8%	0.31
Educational attainment, [*n*, %] Less than high school High school diploma or GED College or Technical School	4, 16.7% 5, 20.8% 15, 62.5%	1, 4.3% 7, 30.4% 15, 65.2%	0.85 ^a^
Employment status, [*n*, %] Working part or full time Retired Disabled or unemployed	8, 33.3% 1, 4.2% 15, 62.5%	4, 17.4% 2, 8.7% 17, 73.9%	0.37 ^b^

^a^ obtained comparing High school diploma/GED or less to Some College/Technical school or more. ^b^ obtained comparing working full- or part-time to retired, disabled or unemployed.

**Table 3:** Focus group themes on the LOVED program.

Themes	Exemplar Quotes
**(1)** **Video chat sessions provided essential support and encouragement**	As the weeks went by, I think we all um… we could say we were, we could consider each other like friends and partners in this uh um war we got going on with kidney disease and I can reach out to any one of these guys in collegiate and just…you know get feedback on something, bounce ideas.” He just stressed the fact that when you ask people you are going to get some “no’s”, but don’t let that…don’t give up on that, keep going, don’t be discouraged. You know, and I appreciate that because that would discourage me, if I go to the first three people and they say “no”, I’m like, “I’m not doing this anymore”, you know, you just give up, you feel like, “nobody is going to help me”, and he just stayed positive the whole time. And I appreciate that. Because if you get so many “no’s” in your face, you’re going to, you know, you will just be like, you just feel discouraged. And he was just very positive, and I appreciated that.” I mean at first I think we all were maybe slightly a little apprehensive…um but as the weeks went by I think we all um… we could say we were, we could consider each other like friends and partners in this uh um war we got going on with kidney disease and I can reach out to any one of these guys in collegiate and just…you know get feedback on something, bounce ideas maybe we can---three of us live kind of really close together, maybe we can get together and do like a big thing together.”
**(2)** **Videos motivated and made me more knowledgeable**	I pretty much enjoyed it because I learned a lot, you know. Before, people would ask me, “Hey how are doing”, and I would just say, “I’m waiting on that call”. You know, for the transplant. And now, I learn information where I can pretty much make it happen myself instead of waiting on the call, and I learned about how to go about doing that, and I learned that there is other people out there with the same situation I’m going through. So I think, everything was good help and good.” I’m a very private person, and for me, I got out this class, there is no reason to be private because everybody is going through the same situation you have, so you might as well be bold about it. And make, do some initiatives, and try to find a kidney for yourself.” I had all of the wrong information and this just gave me a whole, totally different perspective on everything as far as how to go about talking to people about, asking for to be a donor, and just, everything, in general overall.” I um was impressed to learn that uh if a person donates a kidney and later down the line they should need a kidney they go to the top of the list.”
**(3)** **Connectivity with the tablets was adequate in most areas**	I had no problems up until the last session last week. And then tonight, but then, other than that, it was fine. No problems.” The tablet was a good idea. It’s you know, it’s knew technology and um, I like the fact that the instructions you guys gave on how to use the tablet was very simple and easy.” I enjoyed it ’cause I certainly didn’t want to drive back to Charleston. That’s two hours for me. So I enjoyed its convenience. Had to sign for it, no problem. And, the tablet itself was easy. The instructions were very well, if you can read you can get instruction it was very easy, ABC so I enjoyed it. I liked it.”
**(4)** **Material was culturally sensitive**	Yeah I think it could be used for almost anyone because although in the beginning you mentioned in the beginning that it was designed for, to try to help black people living donor, but other than that you could use the same thing and say it’s for, to get uh Hispanic people or white people living donors.”
**(5)** **Being part of a program was overall a positive experience**	I enjoyed the 8 weeks, but I also enjoyed the things that we were doing, and the sessions we were doing, but also what I enjoyed the most was after you finished the session was going to the links and read more about different things, in the links, you know what I am talking about?” I was excited about the program when I got the initial phone call from you about the program. I never heard of the program. I was anxious, so I was anxious, I was excited and then when I received the tablet, I was like, “all right, this is cool”. And then, it was easy to use. I enjoyed meeting everyone. And, I enjoyed the program.” I went back, especially when they did the testimonies and different websites you can go back and see what is going on, and who can help you give you more information about the specifics and the kidney transplant. And all that other kind of stuff, so I read it all the time. That’s the stuff I was telling y’all I was sharing with my family.” The modules were great, they, the videos played without problems…um…it was very easy to use, it was user friendly. I, I didn’t have any problems with accessing the modules, watching the videos, uh looking at the content down below with the brochures and different things. All that was fine. The only problem I had is, maybe just because it was my personal tablet, I could not actually connect on Wednesday night to um…do the video calls. So I just, I opted to use my cell phone and it worked just fine. I had the links downloaded on both.”
**(6)** **Participants were more willing to ask for a kidney now**	It encouraged you to go out and just ask.” I wouldn’t ask beforehand and I was just going to wait for the call, … since the program I’m just like, I try to … approach someone on a day to day basis. It’s like, almost an exemption trying to like get that one person to say yeah. The no’s don’t bother me anymore.” Kind of felt at first that to ask somebody for a kidney, I kind of felt almost like I was being a burden to that person. That I was bothering them and that is just too much to ask of somebody, but um by the end I was feeling a little bit more comfortable to you know, you know already, what does it hurt to ask?” This program was very good to assist us further to approach family and friends to be a live donor.” The program enlightened me more to be able to talk to people about being a live donor.”

**Table 4:** Cultural competency questionnaire results.

Scale	LOVED Mean (SD)	95% CI	Usual Care Mean (SD)	95% CI	*p*-Value
Communication at 8 weeks	3.93 (0.15)	3.86, 3.99	3.78 (0.33)	3.62, 3.93	0.07 *
Communication at 6 months	3.85 (0.31)	3.71, 3.99	3.82 (0.28)	3.68, 3.95	0.71
Trust at 8 weeks	3.95 (0.22)	3.85, 4.05	-	-	-
Trust at 6 months	3.85 (0.31)	3.70, 4.00	-	-	-
Discrimination at 8 weeks	3.96 (0.13)	3.89, 4.02	3.86 (0.36)	3.71, 4.02	0.27 *
Discrimination at 6 months	3.96 (0.15)	3.89, 4.02	3.78 (0.72)	3.42, 4.14	0.32 *
Decision Making at 8 weeks	1.33 (0.67)	1.01, 1.64	-	-	-
Decision Making at 6 months	1.63 (1.03)	1.16, 2.12	-	-	-

ANOVA. * obtained from Welch–Satterthwaite test for unequal variances. Note: Scores represent scale averages on a 1–4 range with higher scores representing positive outcomes except the Decision Making scale, where lower scores represent stronger empowerment.

## Data Availability

Data available on request from the corresponding author due to privacy and ethical conditions restricted by the institution’s IRB.
